# Biological Characteristics of Stromal Cells Differ Between Primary Lung Adenocarcinoma, Peritumoral Lung, and Distal Lung Tissues

**DOI:** 10.1155/sci/6807362

**Published:** 2026-09-07

**Authors:** Go Kamimura, Masaya Aoki, Tao-Sheng Li, Tatsuhiko Furukawa, Masatatsu Yamamoto, Yuka Ishihara, Kohichi Kawahara, Kazuhiro Ueda

**Affiliations:** ^1^ Department of General Thoracic Surgery, Kagoshima University Graduate School of Medical and Dental Sciences, Kagoshima, Japan, kagoshima-u.ac.jp; ^2^ Department of Stem Cell Biology, Atomic Bomb Disease Institute, Nagasaki University, Nagasaki, Japan, nagasaki-u.ac.jp; ^3^ Department of Pathology, Kagoshima University Graduate School of Medical and Dental Sciences, Kagoshima, Japan, kagoshima-u.ac.jp; ^4^ Department of Molecular Oncology, Kagoshima University Graduate School of Medical and Dental Sciences, Kagoshima, Japan, kagoshima-u.ac.jp

**Keywords:** CAFs, distal lung, lung adenocarcinoma, peritumoral lung, stromal cell

## Abstract

**Background:**

Stromal cells are known to play critical roles in the development and progression of solid tumors including lung cancers. This study was proposed to clarify the biological features of stromal cells from intratumoral, peritumoral lung, and distal lung tissues of primary lung adenocarcinoma patients.

**Methods:**

Surgically resected lung lobes containing primary adenocarcinoma were used for isolating stromal cells. Tumor tissue, peritumoral lung tissue, and distal lung tissue were sampled and cut into small pieces for culture as “explants.” The biological features of stromal cells were evaluated by cell isolating efficiency, cell morphology, cell surface marker, gene expression profile, and cytokine secretion.

**Results:**

Spindle shaped fibroblast‐like cells were outgrown successfully from the “explants” with comparable efficiency among different tissues. All outgrowth cells were vimentin‐positive and cytokeratin‐negative, consistent with stromal cell phenotypes. Quantitative analysis on the width‐to‐length ratio of cells indicated different morphological differences among stromal cells. Moreover, the expression of α‐smooth muscle actin (α‐SMA), a marker for cancer‐associated fibroblasts (CAFs), was more extensively observed in stromal cells from tumor tissues than others and, its PCR array showed higher expression of extracellular matrix (ECM)‐related factors *COL11A1* and *TIMP3*, in contrast, cytokine array analysis revealed more abundant release of immune‐suppressive factors from stromal cells of peritumoral tissues.

**Conclusion:**

Stromal cells from tumor, peritumoral, and distal lung tissues display distinct biological characteristics. Further investigation is needed to elucidate their specific roles in tumor progression.

## 1. Background

Malignant tumors contain not only cancer cells but also various extracellular matrix (ECM) and stromal cells that include fibroblasts and immune cells. These stromal cells create a certain microenvironment and play major roles in promoting cancer progression through interactions with cancer cells [1–3]. It is well known that cancer cells release transforming growth factor‐β (TGF‐β) or platelet‐derived growth factor (PDGF) to induce activation of stromal fibroblasts [4–6]. These activated stromal cells may lose intercellular adhesion and adopt a more invasive phenotype, becoming cancer‐associated fibroblasts (CAFs) [7, 8].

CAFs are a major constituent of stromal cells in cancer with various origins and play major roles in tumor growth, angiogenesis, therapeutic resistance, and metastasis. Morphologically, CAFs resemble spindle‐shaped fibroblasts or smooth muscle cells and exhibit mesenchymal characteristics. Consequently, mesenchymal markers such as vimentin are used as positive markers of CAFs, and epithelial markers such as cytokeratin are used as negative markers [9, 10]. Among these, α‐smooth muscle actin (α‐SMA) is the most widely used marker for CAFs.

In contrast to the well‐characterized CAFs, less is known about the stromal cells located in the peritumoral region, which may also influence the cancer progression. Since soluble factors like TGF‐β and PDGF can transform stromal fibroblasts into CAFs, stromal cells in the peritumoral region may exhibit tumor‐promoting traits. Conversely, stromal cells from distant lung tissue are less likely to be influenced by tumor‐secreted factors via paracrine signaling.

In this study, tumor tissue, peritumoral lung tissue, and distant lung tissue were sampled after lung lobectomy and cut into small pieces for culture as “explants.” The morphology, surface markers, and gene expression of the outgrown stromal cells were compared among the three tissue types, as was cytokine production in the culture supernatant. The aim of the present study was to clarify the site‐dependent biological features of stromal cells within the affected lung. These findings may improve our understanding of cancer–stroma interactions and identify novel therapeutic targets.

## 2. Materials and Methods

### 2.1. Patients and Specimens

Curative lobectomy was performed on six female nonsmokers with primary invasive nonmucinous lung adenocarcinoma and no significant medical history. Tumors were staged according to the 8th edition of the TNM Classification for Lung Cancer (TNM‐8) [11]. The clinicopathologic characteristics of each case and the experimental assays performed are summarized in Table 1. Cases 1–3 were used for all analyses except the PCR array analysis, whereas Cases 4–6 were used for the PCR array analysis.

**Table 1:** Clinicopathologic characteristics and experimental assays for each case.

Resected lobe	Case 1	Case 2	Case 3	Case 4	Case 5	Case 6
	Right upper	Right upper	Left upper	Left upper	Left upper	Left lower
Grade	3	2	3	3	1	3
Pl	1	0	0	0	0	0
Ly	0	0	0	0	0	0
V	1	0	1	1	0	1
Pathological stage (TMN‐8)	pT2aN1M0	pT3N0M0	pT1bN0M0	pT1aN0M0	pT1bN0M0	pT1bN0M0
Evaluation of cell extension	〇	〇	〇	—	—	—
Vimentin, cytokeratin, and CD31 ICC	〇	〇	〇	—	—	—
Measurement of cytoplasmic dimensions	〇	〇	〇	—	—	—
PCR array analysis	—	—	—	〇	〇	〇
Cytokine array of culture medium	〇	〇	〇	—	—	—
α‐SMA ICC	〇	〇	〇	—	—	—
Western blot	〇	〇	〇	—	—	—
α‐SMA qPCR	〇	〇	〇	—	—	—
FAP and PDGFRβ qPCR	—	—	〇	—	—	—

*Note:* Clinicopathologic characteristics and experimental assays for each case are summarized in a single table. All patients were female never‐smokers with primary invasive nonmucinous lung adenocarcinoma. 〇 indicates that the assay was performed for that case; blank indicates that it was not.

Abbreviations: ICC, immunocytochemistry; LLL, left lower lobe; LUL, left upper lobe; Ly, lymphatic invasion; pl, pleural invasion; RLL, right lower lobe; RUL, right upper lobe; V, vascular invasion.

### 2.2. Tissue Sampling and Explant Culture

Immediately after the lung lobes were extracted, three tissue samples were collected from each lung lobe.

1. Distal lung: Normal lung tissue >5 cm from the tumor.
2. Peritumoral lung: Normal lung tissue adjacent to the tumor.
3. Tumor: Tumor tissue itself.

**Figure 1:**
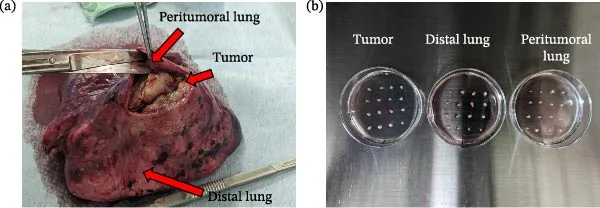
Sampling workflow and explant culture. (a) Tissues were sequentially sampled from distal lung, peritumoral lung, and tumor to prevent cross‐contamination. (b) Minced samples were digested with collagenase IV, inactivated with IMDM + 15% FBS, diced, and plated (16 explants) on fibronectin‐coated dishes.

To avoid cross‐contamination, samples were collected in the order of distal lung, peritumoral lung, and then tumor (Figure 1a). The tissues were cut into 2 mm^3^ pieces and washed five times with phosphate‐buffered saline (PBS) (Cat# 191‐12125, FUJIFILM Wako Pure Chemical Corporation). Enzymatic digestion was performed using collagenase IV (0.2 mg/mL; Cat# LS004186, Worthington Biochemical Corporation) in PBS at 37°C for 30 min. Digestion was terminated by adding Iscove’s Modified Dulbecco’s medium (IMDM) (Cat# 091‐06465, FUJIFILM Wako Pure Chemical Corporation) supplemented with 15% fetal bovine serum (FBS) (Cat# 26140‐079, Thermo Fisher Scientific, Gibco). After further cutting the tissue into 0.5 mm^3^ cubes, 16 pieces were placed onto 6 cm culture dishes precoated with 20 μg/mL fibronectin (Cat# 063–05591, FUJIFILM Wako Pure Chemical Corporation) (Figure 1b). Tissue pieces were immersed in 800 μL of IMDM medium (with 15% FBS) and incubated at 37°C in a humidified atmosphere with 5% CO_2_ for 3 days. Once the tissue pieces adhered, the medium was replaced with 1200 μL of fresh medium every 3–4 days, and the cultures were maintained for 14 days. Adherent cells were then detached using 1000 μL of 0.25% trypsin (Cat# 209‐16941, FUJIFILM Wako Pure Chemical Corporation), followed by dilution with 5000 μL of IMDM (15% FBS) and centrifugation at 300 *g* for 3 min (KUBOTA Centrifuge Model 4000). Subsequent passages were performed at a seeding density of 20% onto 10 cm culture dishes when cells reached 80% confluency, and this procedure was repeated for up to 10 passages.

### 2.3. Evaluation of Cell Extension

**Figure 2:**
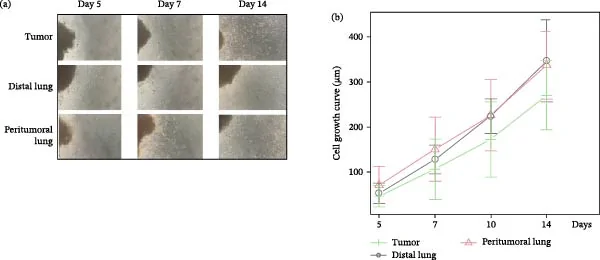
Stromal cell outgrowth and extension. (a) Phase‐contrast images (×40) showing spindle‐shaped cells migrating from tumor, distal lung, and peritumoral lung explants by day 5. (b) Measurement of the three longest extensions (µm) from 16 explants per region; no significant differences among tumor, distal lung, and peritumoral lung.

The extension of cells from tissue explants was evaluated by measuring the distance (in μm) of the three longest continuously extending cell regions from 16 tissue sections (Figure 2a). Statistical analysis was performed using repeated measures analysis of variance (ANOVA) with EZR software (Saitama Medical Center, Jichi Medical University, Saitama, Japan).

### 2.4. Immunocytochemistry for Vimentin, Cytokeratin, CD31, and α‐SMA

For vimentin and cytokeratin staining, passaged‐0 cells (day 7) were fixed with 4% paraformaldehyde. Primary antibodies included anti‐vimentin (Cell Signaling Technology, #P08670; diluted 1:200) and anti‐cytokeratin 19 (Abcam, ab7754; diluted 1:500). Secondary antibodies used were Alexa Fluor 488‐conjugated anti‐rabbit IgG (Cell Signaling Technology, #4412; diluted 1:500) for vimentin and Alexa Fluor 546‐conjugated anti‐mouse IgG (Thermo Fisher Scientific, #A10036; diluted 1:500) for cytokeratin 19. Nuclear staining was achieved using DAPI (DOJINDO, 340‐07971; diluted 1:4000).

To further characterize the explant‐derived stromal cultures and assess possible endothelial cell contamination, CD31 immunocytochemistry was additionally performed using passage 2 cells derived from tumor, peritumoral lung, and distal lung tissues. Cells were fixed with 4% paraformaldehyde and incubated with a ready‐to‐use monoclonal mouse anti‐human CD31 antibody (Agilent, FLEX Monoclonal Mouse Anti‐Human CD31, Endothelial cell, IR610) as the primary antibody. Alexa Fluor 546‐conjugated anti‐mouse IgG (Thermo Fisher Scientific, #A10036; diluted 1:500) was used as the secondary antibody under the same conditions as those described above for cytokeratin 19 staining. Nuclear staining was achieved using DAPI (DOJINDO, 340‐07971; diluted 1:4000). A positive control sample was stained in parallel to confirm the antibody reactivity.

For the immunocytochemical detection of α‐SMA, cells from passages 2, 6, and 10 were fixed with 4% paraformaldehyde for 20 min at room temperature. Following fixation, cells were permeabilized with PBST (0.3% Triton X‐100 in PBS) for 60 min at room temperature and then blocked with blocking buffer (5% bovine serum albumin [BSA] in PBST) for 60 min. The cells were subsequently incubated overnight at 4°C with a monoclonal α‐SMA primary antibody (Proteintech, Cat# 67735‐1‐lg) diluted 1:400 in a blocking solution. In parallel, negative control samples were prepared by replacing the primary antibody with a 5% BSA solution. After three washes with PBS, cells were incubated for 1 h at room temperature in the dark with an Alexa Fluor 546‐conjugated goat anti‐mouse IgG secondary antibody (Thermo Fisher Scientific, Cat# A‐11003) diluted 1:500 in blocking buffer. Fluorescence images were acquired on a Keyence BZ‐×700 all‐in‐one fluorescence microscope (Keyence Corp., Osaka, Japan) equipped with a 40× Plan Apo objective (NA 0.95) and standard filter cubes for DAPI (excitation 360 ± 20 nm and emission 460 ± 25 nm), Alexa Fluor 488 (excitation 470 ± 20 nm and emission 525 ± 25 nm), and Alexa Fluor 546 (excitation 540 ± 12 nm and emission 605 ± 35 nm). Exposure times were 50 ms for DAPI, 100 ms for Alexa Fluor 488, and 150 ms for Alexa Fluor 546. Bright‐field images were also acquired on the same microscope under transmitted‐light conditions.

### 2.5. Measurement of Cytoplasmic Dimensions

For evaluation of the cytoskeletal structure, the width and length of the cytoplasm in spindle‐shaped cells extending from tissue explants were measured under a fluorescent microscope at 40× magnification. Ten randomly selected cells per field were measured across three fields for each region. Similar measurements were performed on spindle‐shaped cells in passage 2. Comparisons among the three regions were analyzed using the Steel–Dwass test in EZR software.

### 2.6. PCR Array Analysis

Spindle‐shaped cells that extended directly from tissue explants from Cases 4–6 were used for PCR array analysis. Total RNA was isolated using the RNeasy Mini Kit (QIAGEN) according to the manufacturer’s instructions, and first‐strand cDNA was synthesized with the ReverTra Ace qPCR RT Master Mix (TOYOBO, FSQ‐301). Gene expression profiling was performed using the RT^2^ Profiler PCR Array Human ECM and Adhesion Molecules (QIAGEN, PAHS‐013 Z, Cat. Number 330231). Quantitative PCR was performed on an Applied Biosystems QuantStudio Real‐Time PCR System (Thermo Fisher Scientific) under the following cycling conditions: 95°C for 2 min, followed by 40 cycles of 95°C for 15 s, and 60°C for 30 s and a melt‐curve analysis of 95°C for 15 s, 60°C for 1 min, and 95°C for 15 s. RNase‐free water served as the no‐template control, and all reactions were carried out in triplicate. Relative expression levels of each gene in tumor and peritumor samples were calculated using distal lung samples as the calibrator.

Statistical analyses were conducted using the EZR software. Homogeneity of variances among CNTN1, COL11A1, and TIMP3 expression levels was first assessed by the *F*‐test. CNTN1 expression differences were then evaluated using Welch’s *t*‐test, while differences in COL11A1 and TIMP3 expression were assessed by the Student’s *t*‐test.

### 2.7. Cytokine Array of Culture Medium

The culture medium was collected from passage 1 cells at 80% confluency from each of the three regions (distal lung, peritumoral lung, and tumor) for Cases 1–3. Medium samples from each region were pooled for each case, and cytokine levels were measured using the Proteome Profiler Human Angiogenesis Array Kit (Bio‐Techne, R&D Systems) following the manufacturer’s instructions. Dot plot images were captured using the MultiImager Ⅱ ChemiBOX (BioTools) and analyzed using ImageJ software. Cytokines with an integrated density (IntDen) value ≥ 500 were recorded.

### 2.8. Western Blot

Proteins were extracted from cultured cells after washing with PBS and homogenizing in lysis buffer (protease inhibitor cocktail [Cat#165‐26021, FUJIFILM Wako Pure Chemical Corporation] diluted 1:100 in RIPA buffer [Cat#182‐02451, FUJIFILM Wako Pure Chemical Corporation]), then centrifuged (15,000 rpm, 30 min, 4°C). Protein concentration was determined by a Bradford assay; 15 µg per lane was denatured in 4× sample buffer (4× Laemmli sample buffer [Bio‐Rad, Cat# 1610747] supplemented with 10% [v/v] 2‐mercaptoethanol) at 100°C for 5 min, separated by sodium dodecyl sulfate–polyacrylamide gel electrophoresis (SDS–PAGE), and transferred to polyvinylidene difluoride (PVDF) membranes using a Trans‐Blot Turbo system (BIO RAD 1.0 A, 20 V, 30 min). Membranes were blocked in 3% BSA in Tris‐buffered saline with 0.1% Tween‐20 (TBST) for 30 min and then incubated with an anti‐α‐SMA (Proteintech, Cat# 67735‐1‐lg; diluted 1:20,000) primary antibody for 60 min, followed by an HRP‐linked anti‐mouse IgG secondary antibody (Cellsignaling #7076 diluted 1:2000) for 60 min. Bands were visualized using enhanced chemiluminescence (ECL) (BIO RAD). Chemiluminescent signals were captured using the MultiImager Ⅱ ChemiBOX. For the loading control, membranes were stripped and reprobed with an anti‐GAPDH (Cell Signaling, #2118 diluted 1:500) primary antibody and an HRP‐linked anti‐rabbit IgG secondary antibody (Cellsignaling, #7074 diluted 1:2000). Densitometric analysis of chemiluminescent bands was performed using ImageJ software (NIH). Band intensities for α‐SMA were normalized to the corresponding GAPDH signal. Statistical comparisons among tumor, peritumor, and distal‐lung groups were performed using the Steel–Dwass test in EZR software.

### 2.9. α‐SMA, FAP, and PDGFRβ qPCR

Total reaction volumes were 20 µL, prepared by combining 10 µL of Luna Universal qPCR Master Mix (2×; New England BioLabs), 0.5 µL each of α‐SMA‐specific forward and reverse primers (10 µM), 8 µL of RNase‐free water, and 3 µL of the cDNA template. Primers were designed using Primer3 [12], and the sequences for α‐SMA were: forward 5^′^‐CACCACTATGTACCCTGGCA‐3^′^ and reverse 5^′^‐CCGGCTTCATCGTATTCCTG‐3^′^ (each 20 bp). Negative controls were set up by replacing cDNA with RNase‐free water. Reactions were run on an Applied Biosystems StepOnePlus instrument under the following conditions: initial denaturation at 95°C for 2 min; 40 cycles of 95°C for 15 s and 60°C for 30 s; followed by melt curve analysis (95°C for 15 s, 60°C for 1 min, and 95°C for 15 s). GAPDH was used as the internal control for normalization.

Relative α‐SMA gene expression ratios of tumor and peritumoral lung samples were calculated against lung samples at both passage 2 and passage 10. Statistical comparisons among the three groups were performed using the Steel–Dwass test in EZR software. To further characterize stromal heterogeneity, transcript expression of FAP and PDGFRβ was additionally evaluated in passage 2 stromal cells derived from Case 3. For each region (tumor, peritumoral lung, and distal lung), qPCR was performed in technical triplicate using the same sample, and the mean values are shown in Supporting Information 1: Figure S2. Total RNA extraction, cDNA synthesis, and quantitative PCR were performed as described above. Because these supplementary data were obtained from a single case and measured in technical triplicate, they are presented descriptively and were not used for formal statistical comparison among biologically independent groups.

## 3. Results

### 3.1. Morphological Analysis of Stromal Cells

**Figure 3:**
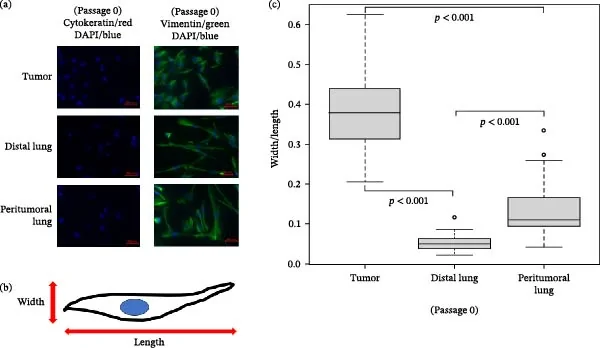
Stromal cell marker expression and cytoplasmic morphology. (a) All explant‐derived cells (tumor, distal lung, and peritumoral lung) stained positive for vimentin and negative for cytokeratin‐19 (green/red; nuclei blue). (b) Schematic of cytoplasmic width and length measurements in spindle‐shaped cells. (c) Tumor cells displayed thicker, shorter cytoplasm, distal lung cells were thinner and elongated, and peritumoral cells showed intermediate morphology; width to length ratios differed significantly among regions (*p* < 0.001).

Microscope images (×40) of explants from tumor, peritumoral lung, and distal lung regions are shown in Figure 2a. Spindle‐shaped cells emerged from all tissue types starting on day 5, with no significant differences in the rate of extension between the regions (tumor vs. distal lung, *p* = 0.139; tumor vs. peritumoral lung, *p* = 0.566; distal lung vs. peritumoral lung, *p* = 0.707; Figure 2b). Immunocytochemical staining demonstrated that all cells were vimentin‐positive and cytokeratin‐negative, confirming their stromal origin (Figure 3a). In addition, CD31 immunocytochemistry showed no detectable CD31‐positive cells in passage 2 cultures derived from tumor, peritumoral lung, or distal lung tissues, whereas the positive control showed clear staining (Supporting Information 1: Figure S1). These findings suggest that overt endothelial cell contamination was minimal under the present culture conditions.

Cytoplasmic dimension measurements (Figure 3b) showed that tumor‐derived stromal cells had thicker and shorter cytoplasm, distal lung stromal cells were thinner and more elongated, and peritumoral stromal cells exhibited intermediate features. Statistical analysis revealed significant differences among regions (*p*  < 0.001 for all comparisons) (Figure 3c). This result supports that peritumoral lung stromal cells have different characteristics from either tumor stromal cells or distal lung stromal cells. In passage 2 cells, a tendency toward a decreased width to length ratio was observed in the tumor and peritumoral lung stromal cells, although these differences were not statistically significant (tumor P0 vs. P2, *p* = 0.109; peritumoral lung P0 vs. P2, *p* = 0.186), while no change was observed in distal lung stromal cells (P0 vs. P2, *p* = 0.751).

These results suggested that peritumoral lung stromal cells might have different characteristics from tumor stromal cells and distal lung stromal cells.

### 3.2. Gene Expression Profiling

**Figure 4:**
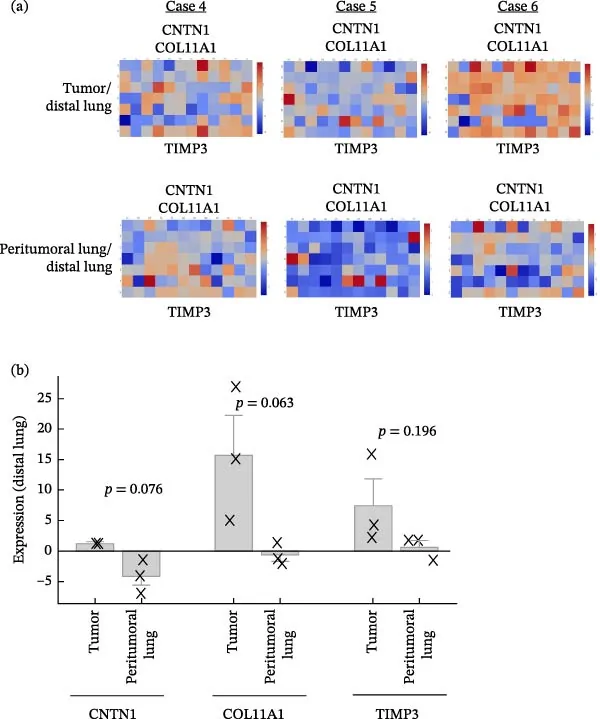
Differential gene expression in stromal cells. (a) Heatmap of PCR array‐derived relative fold changes in tumor and peritumoral lung stromal cells compared with distal lung stromal cells from Cases 4–6. (b) Relative expression of selected genes of interest. *COL11A1* and *TIMP3* were higher in tumor stromal cells, whereas *CNTN1* was lower in peritumoral lung stromal cells. *CNTN1* and *COL11A1* showed a trend toward differential expression.

PCR array analysis of stromal cells from Cases 4–6 demonstrated that *COL11A1* and *TIMP3* expression was higher in the tumor region compared to both distal lung and peritumoral lung regions. Conversely, the expression of *CNTN1* was lower in the peritumoral lung region than in the other two regions (Figure 4a, Supporting Information 2: Table S1). The gene names corresponding to the heatmap are provided in Supporting Information 1: Figure S3. Despite the limited sample size, both *CNTN1* and *COL11A1* exhibited trends toward differential expression (tumor/distal lung vs. peritumoral lung/distal lung; *CNTN1*: *p* = 0.076, *COL11A1*: *p* = 0.063, and *TIMP3*: *p* = 0.196) (Figure 4b).

### 3.3. Protein Secretion Patterns

**Figure 5:**
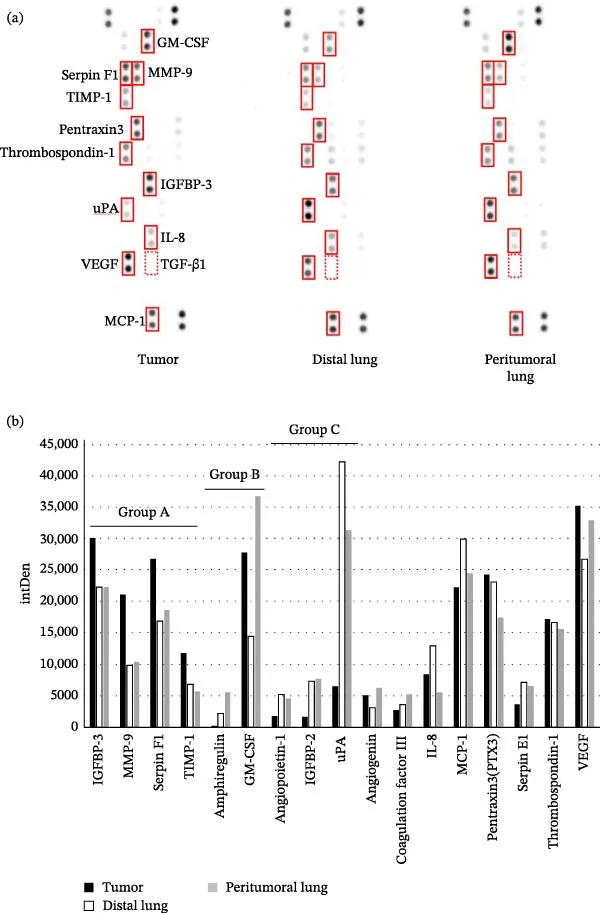
Cytokine secretion profiles of stromal cells. (a) Representative dot‐blot array images for passage 1 culture supernatants from tumor, distal lung, and peritumoral lung stromal cells. (b) Quantified intensity patterns (via ImageJ) categorizing cytokines into: Group A: higher expression in tumor stromal cells (IGFBP‐3, MMP‐9, Serpin F1, and TIMP‐1), Group B: higher expression in peritumoral lung stromal cells (amphiregulin, GM‐CSF), and Group C: lower expression in tumor stromal cells (angiopoietin‐1, IGFBP‐2, and uPA).

Analysis of the culture medium from passage 1 cells (via dot plot and ImageJ analysis) revealed distinct protein expression patterns (Figure 5a,b and Supporting Information 2: Table S2). Cytokines were classified into three groups as follows:

1. Group A: Higher expression in tumor stromal cells (e.g., IGFBP‐3, matrix metalloprotein [MMP]‐9, Serpin F1, and TIMP‐1).
2. Group B: Higher expression in peritumoral lung stromal cells (e.g., amphiregulin and GM‐CSF).
3. Group C: Lower expression in tumor stromal cells (e.g., angiopoietin‐1, IGFBP‐2, and uPA).

Tumor stromal cells exhibited high expression of IGFBP‐3, MMP‐9, Serpin F1, and TIMP‐1 and low expression of angiopoietin‐1, IGFBP‐2, and uPA. The expression of amphiregulin and GM‐CSF was high in peritumoral lung stromal cells. These results support that peritumoral lung stromal cells have different characteristics from tumor stromal cells and distal stromal cells.

### 3.4. α‐SMA FAP and PDGFRβ Expression

**Figure 6:**
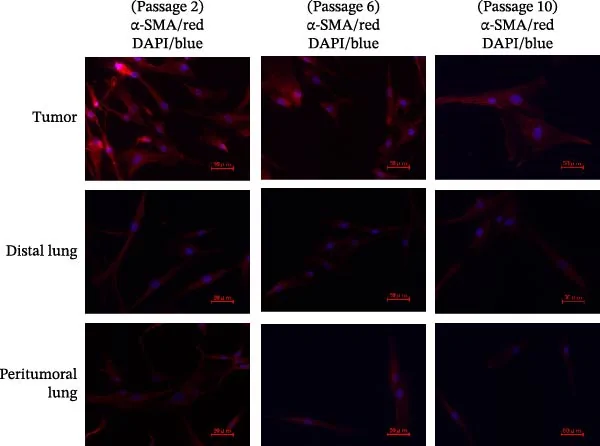
α‐SMA immunostaining of stromal cells. Passage 2 tumor stromal cells show prominent α‐SMA staining (red), whereas distal lung and peritumoral lung stromal cells show weaker staining; nuclei in blue (DAPI).

**Figure 7:**
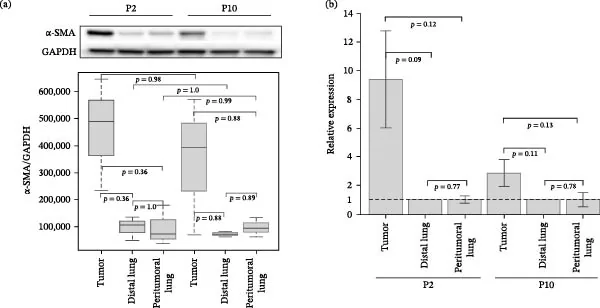
Passage‐dependent α‐SMA expression in stromal cells. (a) Western blots show high α‐SMA protein in tumor stromal cells at passage 2, decreasing by passage 10, compared to distal lung and peritumoral lung. (b) Densitometry and qPCR confirm elevated α‐SMA in tumor P2 with a decline at P10.

To assess whether peritumoral stromal cells exhibit distinct activation states, α‐SMA expression was evaluated. Immunocytochemistry results showed that α‐SMA staining was most prominent in tumor stromal cells at passage 2, whereas distal lung and peritumoral lung stromal cells showed weaker staining (Figure 6). Western blot analysis confirmed that α‐SMA protein levels were higher in tumor stromal cells at passage 2 compared to distal lung stromal cells and peritumoral lung stromal cells. However, by passage 10, α‐SMA expression in tumor stromal cells had decreased (Figure 7a). Quantification using ImageJ indicated a tendency toward higher α‐SMA expression in tumor stromal cells at passage 2 (tumor P2 vs. distal lung P2, *p* = 0.36; tumor P2 vs. peritumoral lung P2, *p* = 0.36), with a slight decrease observed upon extended passaging (tumor P2 vs. P10, *p* = 0.98) (Figure 7a). Real‐time PCR analysis showed that α‐SMA mRNA expression (normalized to distal lung stromal cells at the same passage) was higher in tumor stromal cells at both passage 2 (tumor P2 vs. distal P2, *p* = 0.09; tumor P2 vs. peritumoral P2, *p* = 0.12) and passage 10 (tumor P10 vs. distal P10, *p* = 0.11; tumor P10 vs. peritumoral P10, *p* = 0.13), although expression levels declined over time (Figure 7b). These results indicate that peritumoral lung stromal cells have different characteristics from tumor stromal cells. In addition, the observation that the expression of α‐SMA, CAF marker, decreased gradually in tumor stromal cells with passage suggested that the features of tumor stromal cells depend on the presence of cancer cells.

To further evaluate stromal heterogeneity beyond α‐SMA alone, transcript expression of FAP and PDGFRβ was additionally assessed in passage 2 stromal cells from Case 3 (Supporting Information 1: Figure S2). These supporting exploratory data showed relatively higher FAP expression in tumor stromal cells than in distal lung stromal cells, whereas PDGFRβ expression appeared higher in tumor stromal cells and lower in peritumoral lung stromal cells relative to distal lung stromal cells.

## 4. Discussion

CAFs are mainly derived from stromal fibroblasts in normal lung and acquire their biological characteristics through crosstalk with cancer cells via soluble factors within the tumor microenvironment [9, 13]. However, tumor‐derived soluble factors may also influence stromal cells outside the tumor. Based on this, we hypothesized that stromal cells in the peritumoral lung might exhibit biological characteristics distinct from those of both intratumoral and distal lung stromal cells. In this study, we compared the biological characteristics of stromal cells derived from intratumoral, peritumoral, and distal lung tissues in primary lung adenocarcinoma. As expected, each stromal cell population exhibited distinct characteristics. Notably, peritumoral stromal cells did not show an intermediate phenotype between intratumoral and distal lung stromal cells.

Unlike intratumoral stromal cells, peritumoral stromal cells did not show elevated expression of ECM‐related genes and exhibited limited α‐SMA expression. Additional evaluation of FAP [14, 15] and PDGFRβ [14, 15] further supported stromal heterogeneity: FAP tended to be higher in tumor stromal cells than in distal lung stromal cells, whereas PDGFRβ tended to be higher in tumor stromal cells and lower in peritumoral lung stromal cells relative to distal lung stromal cells. In contrast, peritumoral stromal cells showed a relatively higher expression of immunomodulatory factors. Taken together, these findings suggest that stromal cells from the three regions may differ in stromal composition and/or activation state rather than representing a simple continuum of a single uniform population.

Immunocytochemistry of cultured cells showed that all three stromal populations were consistently vimentin‐positive and cytokeratin‐negative, indicating their mesenchymal origin. Because explant‐derived cultures may potentially include multiple nonimmune stromal cell types, we additionally evaluated the CD31 expression to assess endothelial cell inclusion. No CD31‐positive cells were detected in the examined cultures from tumor, peritumoral lung, or distal lung tissues, suggesting that the observed regional differences were unlikely to be primarily driven by endothelial contamination. In contrast, α‐SMA expression was more prominent in intratumoral stromal cells than in peritumoral or distal lung stromal cells, suggesting a difference in activation state. The present explant‐derived cultures should be interpreted as bulk stromal outgrowth cultures rather than fully purified stromal subsets. Interestingly, the expression level of α‐SMA seemingly weakened by 10 times passage culturing of intratumoral stromal cells, which was in accordance with a previous report on the results of passage culturing of CAFs isolated from ovarian cancer tissue [16]. This observation supports the notion that continuous interaction with cancer cells is necessary to maintain CAF‐like features. In contrast to α‐SMA expression, cytoskeletal morphology varied significantly among the three stromal populations, with peritumoral stromal cells displaying intermediate cytoplasmic thickness between intratumoral and distal stromal cells. We expect the morphology of the three stromal cell types to be similar by continued passage culturing without cancer cells. These results may suggest that the current analysis with cells at passage 2 was valid.

PCR array results revealed higher expression of COL11A1 and TIMP3 in the intratumoral stromal cells than in distal site stromal cells, while the expression level of COL11A1 and TIMP3 in peritumoral stromal cells was comparable to that in distal site stromal cells. COL11A1 is specifically activated in CAFs and is implicated in promoting epithelial–mesenchymal transition (EMT), invasion, and metastasis [17]. In contrast, the TIMP3 protein binds to MMPs and inhibits their activity, thereby preventing the degradation of the ECM and suppressing cancer invasion [18]. These conflicting results were also found in the cytokine array analysis of the conditioned medium: both tumor‐promoting protein (MMP‐9) [19] and tumor‐preventing protein (IGFBP‐3 [20], Serpin F1 [21], and TIMP‐1 [22, 23]) were highly expressed in media from intratumoral stromal cells, as compared with that from distal site stromal cells, and tumor‐promoting proteins (Angiopoietin‐1 [24], IGFBP‐2 [25, 26], and uPA [27, 28]) were decreased in media from intr‐tumoral stromal cells. These proteins directly or indirectly act on the ECM [22, 23, 25–28] and are involved in its degradation and remodeling, thereby controlling cancer proliferation, invasion, and metastasis by participating in angiogenesis [21, 25, 26], apoptosis [20], and cell–cell adhesion [24]. However, these proteins can interact with each other, which eventually contribute to promoting or preventing tumor progression. For instance, tumor‐promoting protein, MMP‐9, can degrade tumor‐preventing cytokine, IGFBP‐3, which binds to the ECM and is involved in the inhibition of proliferation and the induction of apoptosis of cancer cells [29]. Importantly, the levels of these tumor‐promoting or preventing factors expressed by peritumoral stromal cells were comparable to those expressed by distal stromal cells, suggesting that peritumoral stromal cells do not predominantly display a myofibroblastic CAF‐like phenotype.

Despite not expressing classical CAF markers or ECM remodeling factors, peritumoral stromal cells may still facilitate tumor progression through other mechanisms. In fact, our PCR array analysis revealed that CNTN1 [30]—a cell‐adhesion molecule implicated in EMT and tumor invasion—was expressed at the lowest level in peritumoral stromal cells compared with both intratumoral and distal lung stromal cells. In contrast, cytokine array analysis revealed that amphiregulin and GM‐CSF levels were the highest in peritumoral stromal cells. Amphiregulin has been implicated in regulatory T‐cell–mediated immunosuppression and tumor progression [31], while GM‐CSF promotes immune evasion via tumor‐associated macrophages and enhances proliferation and metastasis [32]. These results suggest that peritumoral stromal cells may contribute to tumor progression by modulating the immune microenvironment. Our hypothesis is supported by a previous study conducted by Itoh et al. [33] which reported that soluble factors from CAFs, but not tumor cells, induce phenotypic change in normal fibroblasts. This process does not lead to α‐SMA upregulation but rather results in increased expression of inflammatory cytokines, which in turn contributes to the suppression of CD8+ T cells [33]. They suggested that normal fibroblasts can be directly educated by CAFs to facilitate tumor progression. Whether or not the peritumoral stromal cells in our study were influenced by CAFs should be clarified by the next study with CAFs and normal fibroblasts.

This study has several limitations. First, stromal cell phenotypes may vary among patients depending on the underlying lung conditions or cancer stage. The biological aggressiveness of CAFs may also differ across histological subtypes. Therefore, the properties of peritumoral stromal cells may not be uniform. Larger‐scale studies across diverse patient populations and disease stages are required. Second, the current study evaluated the global change of various soluble factors in each stromal cell group. According to previous studies, there are some subtypes in CAFs, including tumor‐promoting and tumor‐preventing CAFs [9, 13]. Further evaluation of heterogeneity in stromal cells is needed to better understand the peritumor microenvironment. In addition, the present study did not include tissue‐based spatial validation of the explant culture findings by immunohistochemistry or in situ hybridization. Therefore, further studies are needed to confirm whether the regional differences observed in the cultured stromal cells are recapitulated within the histologic spatial context of the tumor.

## 5. Conclusion

Peritumoral stromal cells exhibited biological characteristics distinct from those of intratumoral stromal cells and did not predominantly display a myofibroblastic CAF‐like phenotype. These findings underscore the heterogeneity of stromal populations within the tumor microenvironment and suggest that peritumoral stromal cells may contribute to tumor progression through alternative mechanisms. Further research is needed to elucidate the unique peritumor stromal cell microenvironment we have newly identified.

## Author Contributions

Go Kamimura, Tao‐Sheng Li, and Kazuhiro Ueda conceived and designed the study. Go Kamimura, Yuka Ishihara, Masaya Aoki, and Kohichi Kawahara collected the data. Go Kamimura, Tatsuhiko Furukawa, and Masatatsu Yamamoto performed the data analysis. Go Kamimura, Tao‐Sheng Li, and Kazuhiro Ueda drafted the manuscript.

## Funding

This work was supported by the Japan Society for the Promotion of Science (JSPS) KAKENHI Grant‐in‐Aid for Scientific Research (C), “Exploration of Target Molecules Based on Comprehensive Analysis of Functional Acquisition Mechanisms of Cancer‐Associated Stromal Cells” (Grant 20K09182) and Research Support Project for Life Science and Drug Discovery (Basis for Supporting Innovative Drug Discovery and Life Science Research (BINDS)) from Japan Agency for Medical Research and Development (AMED) (Grant JP24ama121054).

## Disclosure

All authors read and approved the final manuscript.

## Ethics Statement

This study was approved by the Kagoshima University Hospital Ethics Committee (Approval Number 200304).

## Consent

Consent has been obtained from all concerned parties. All participants provided written informed consent prior to enrollment.

## Conflicts of Interest

The authors declare no conflicts of interest.

## Supporting Information

Additional supporting information can be found online in the Supporting Information section.

## Supporting information


**Supporting Information 1** Figure S1. CD31 immunocytochemistry of explant‐derived stromal cultures. Representative bright‐field and immunofluorescence images of passage 2 cells derived from tumor, peritumoral lung, and distal lung tissues are shown. No detectable CD31‐positive cells (red) were observed in the examined cultures, whereas the positive control demonstrated clear CD31 staining. Nuclei were counterstained with DAPI (blue). Figure S2. Exploratory evaluation of FAP and PDGFRβ expression in stromal cells from Case 3. (a) Relative FAP mRNA expression in passage 2 stromal cells derived from tumor, distal lung, and peritumoral lung tissues from Case 3. (b) Relative PDGFRβ mRNA expression in passage 2 stromal cells derived from tumor, distal lung, and peritumoral lung tissues from Case 3. For each region, qPCR was performed in technical triplicate, and mean values are shown. Figure S3. Gene names and sample IDs corresponding to the heatmap in Figure 4a. The gene names corresponding to the heatmap shown in Figure 4a are indicated in the figure.

**Supporting Information 2** Table S1. PCR array fold‐changes for all genes. Lists fold‐change values of each gene measured by RT^²^ Profiler PCR Array in tumor stromal cells and peritumoral lung stromal cells relative to distal lung stromal cells for Cases 4–6. Table S2. Cytokine dot‐blot integrated densities mean ImageJ–determined integrated density (IntDen) values for all cytokines detected in conditioned medium of passage 1 stromal cells from tumor, peritumoral lung, and distal lung regions (Cases 1–3).

## Data Availability

The data that support the findings of this study are available from the corresponding author upon reasonable request.
